# Nonpharmacological Interventions for Pain in Patients with Temporomandibular Joint Disorders: A Systematic Review

**DOI:** 10.1055/s-0041-1740220

**Published:** 2022-03-08

**Authors:** Liliana Argueta-Figueroa, Luis Angel Flores-Mejía, Beatriz Xóchitl Ávila-Curiel, Blanca Irma Flores-Ferreyra, Rafael Torres-Rosas

**Affiliations:** 1 CONACyT – Facultad de Odontología, Universidad Autónoma “Benito Juárez” de Oaxaca, Oaxaca, México; 2 Sponsored Researcher, School of Public Health, Imperial College, London, United Kingdom; 3 Laboratorio de Medicina Complementaria, Centro de Estudios en Ciencias de la Salud y la Enfermedad, Facultad de Odontología, Universidad Autónoma “Benito Juárez” de Oaxaca Oaxaca, México; 4 Facultad de Odontología, Universidad Autónoma del Estado de México (UAEMex), Toluca de Lerdo, México

**Keywords:** dentistry, physiotherapy, complementary, pain, temporomandibular joint

## Abstract

This systematic review aimed to compare the efficacy of nonpharmacological therapies for painful temporomandibular joint disorders. The protocol was registered on International Prospective Register of Systematic Reviews (PROSPERO) database (CRD42020171364). The search was performed on the electronic databases PubMed, Google Scholar, Clinical Trials, and Web of Science. The eligibility criteria were randomized controlled trials in patients diagnosed with painful temporomandibular joint disorders comparing the pain relief between conventional treatment and nonpharmacological therapies such as acupuncture, physiotherapy, low-level laser, and massage. Fourteen articles were included in this review. At the overall bias of the studies included, 71.42% exhibited some concerns and 28.57% had high risk. The efficacy of nonpharmacological interventions was found to be moderate in the short term and variable in the long term for pain reduction in patients with temporomandibular joint disorders. The evidence pointed out that acupuncture, laser therapy, and physiotherapy are potentially useful interventions for pain relief in patients with temporomandibular joint disorders. However, there is a lack of consistency and short-term follow-up in the studies to determine the lasting of such effect.

## Introduction

The temporomandibular joint (TMJ), the jaw, the muscles, the ligaments, the periodontium, and the dental organs complete a functional stomatognathic unit. Disruption of those components derives into homeostatic rupture known as temporomandibular disorders (TMD) with the development of pain and mandibular movement alterations. 1 2 These disorders include a highly heterogeneous group of clinical conditions characterized by pain and dysfunction of the masticatory system. 3 4

TMD are frequent, and even one in ten patients with TMD has been suffered from severe pain-related disability, 5 which directly affects their quality of life. 6 It has been reported that in TMD, there is an increased risk of presenting a greater number of painful sites as well as coexisting pain and comorbidities when the duration of pain is increased. This pain is probably attributable to the central sensitization mechanism, which is time-dependent. That disorder can evolve to chronicity if proper treatment is not provided. Hence, avoiding chronic pain through timely and efficient treatment is important. 7

The Diagnostic Criteria for Temporomandibular Disorders (DC/TMD) is a validated instrument for diagnosis and research of TMD, which is divided into two axes. Axis I is diagnostic, while Axis II is about behavioral factors. Axis I shows a taxonomic classification as (1) temporomandibular joint disorders (TMJD), (2) masticatory muscle disorders, (3) headache, and (4) associated structures. TMJ disorders are divided into (1) joint pain, (2) joint disorders, (3) joint diseases, (4) fractures, and (5) congenital/developmental disorders.

The treatment of TMJDs is complex depending on the severity of the pathology and the structures affected. 8 These disorders often require a complete pharmacological scheme that includes anxiolytics, analgesics, muscle relaxants, and in some cases, even antidepressants. However, these treatments frequently have considerable adverse effects, making nonpharmacological approaches potentially advisable options for pain treatment. 9

## Methods

### Protocol Registry

The protocol was registered (CRD42020171364) in the International Prospective Register of Systematic Reviews (PROSPERO).

### Study Design

The types of included studies in the systematic review were controlled trials and were excluded case reports, case series, letters, comments, short communications, pilot studies, animal studies, *in vitro* studies, and literature reviews.

### Eligibility Criteria and Participant Characteristics of Studies

The eligibility criteria were defined considering the PICO (population, intervention, comparison, and outcome) definitions as follows:

Population—The population of included studies must be patients with TMJD (joint pain, joint disorders, and joint diseases). However, studies that included patients with fractures and congenital/developmental disorders (aplasia, hypoplasia and hyperplasia) and patients with diagnoses of masticatory muscle disorders were excluded. The included studies preferably should use the DC/TMD from the International RDC/TMD Consortium Network workshop.
Interventions—The included studies must use the following interventions for pain relief of TMJD:

(1) Acupuncture (AT): The intervention consisting of AT on corporeal points and not microsystems such as hand, foot, and ear system, with traditional AT placement (needle puncture with manual stimulation or electroacupuncture) for at least four sessions. Moxibustion, laser applied at AT points, fire needle, among others were excluded.
(2) Physiotherapy (PT): The intervention must be consisting of jaw exercises for at least 3 weeks. However, PT combined with other interventions such as ultrasound, AT or laser therapy (LT) was excluded.
(3) Low-level laser: The intervention must be low-lever laser therapy (LLLT) for at least four sessions applied on the joint zone or the masticatory muscles were included. The therapy without a full description of the application site, time, dose, sessions, and characteristics of the equipment was excluded.
(4) Massage—The characteristics of the massage were stroking or light pressure manual massage for at least four sessions. Also, massage that consisted of mobilizing joint, passive traction, and translation movements of the jaw was included. Other kinds of massage, such as percussion, vibration, or mechanical massage, were excluded.

Comparator—The included studies must use occlusal splint (OS), pharmacotherapy (PY), or placebo (PO) as the control group. The studies without a control group established in the eligibility criteria (using the same intervention with some variant, for example) or without a PO, as well as other therapies, were not included in the review.

Outcome—Pain relief of TMJD was defined as a change in the pain level score attributable to any intervention described above, from the baseline to the end of the follow-up. The outcome must be assessed by the visual analog scale (VAS).

### Search Strategy and Databases Used

The algorithms used for the search strategy are shown in Table 1 . The search was performed on the electronic databases PubMed, Google Scholar, ClinicalTrials, and Web of Science by two reviewers (RTR and LAF). The manual search was performed through the examination of the reference list from the included studies in the review. The search for gray literature was performed on Google Scholar.

**Table 1:** Keywords used in the search identify through PICO strategy

Population	Patients with temporomandibular joint disorders
Intervention	Acupuncture, physiotherapy, laser, or massage
Comparator	Placebo, occlusal splints or pharmacologic treatment
Outcomes	Pain
Study design	Clinical trials
Electronic database	Medline/PubMed, Google Scholar, Clinical Trails.gov, Web of Science
Focused question	What is the most effective non-pharmacological therapy for pain relief in patients with temporomandibular join disorders?
Number of registers found for each database Accessed: 22/09/2021	Algorithms used for search strategy adapted for each database
PubMed: 734	((“acupuncture”[MeSH Terms] OR “acupuncture”[All Fields] OR “acupuncture therapy”[MeSH Terms] OR (“acupuncture”[All Fields] AND “therapy”[All Fields]) OR “acupuncture therapy”[All Fields]) OR “occlusal splint”[All Fields] OR (“physical therapy modalities”[MeSH Terms] OR (“physical”[All Fields] AND “therapy”[All Fields] AND “modalities”[All Fields]) OR “physical therapy modalities”[All Fields] OR “physiotherapy”[All Fields]) OR (“lasers”[MeSH Terms] OR “lasers”[All Fields] OR “laser”[All Fields]) OR (“massage”[MeSH Terms] OR “massage”[All Fields]) OR (“exercise”[MeSH Terms] OR “exercise”[All Fields])) AND (“Temporomandibular joint disorders”[All Fields] OR “temporomandibular disorders”[All Fields] OR “Temporomandibular joint dysfunction syndrome”[All Fields]) AND (“Pain”[MeSH Terms] OR “pain measurement”[All Fields] OR “visual analogue scale”[All Fields] OR “VAS”[All Fields] OR “pain relief”[All Fields])
Google Scholar: 2,100	“Clinical trial” + (Acupuncture OR “occlusal splint” OR physiotherapy OR laser OR massage) + (“Temporomandibular joint disorders” OR “temporomandibular disorders” OR “Temporomandibular joint dysfunction syndrome”)”) + (“pain measurement” OR “visual analogue scales for pain” OR “VAS” OR “pain relief”)
Clinical Trials: 7	Completed Studies | Studies With Results | Interventional Studies | “Temporomandibular joint disorders” OR “temporomandibular disorders” OR “Temporomandibular joint dysfunction syndrome” | occlusal splint OR physiotherapy OR laser OR massage OR Acupuncture | Pain | Adult | Results first posted Applied Filters: Completed, With Results
Web of Science: 725	TS= (Acupuncture OR “occlusal splint” OR physiotherapy OR laser OR massage OR exercise) TS = (“Temporomandibular joint disorders” OR “temporomandibular disorders” OR “Temporomandibular joint dysfunction syndrome”) Indexes = SCI-EXPANDED, SSCI, A&HCI, CPCI-S, CPCI-SSH, BKCI-S, BKCI-SSH, ESCI Timespan = All years

### Study Selection

The process of selecting studies was performed by two reviewers (ACBX and FFBI). A third reviewer (RTR) resolved the disagreements. The eligibility of the studies that could be included in the review was determined by reading the title and summary of each record identified in the search. The full-text of the studies that met the eligibility criteria for in-depth review was then retrieved. After reviewing the full-text, if the studies did not fully meet the eligibility criteria, these were excluded with reasons.

### Data Collection Process and Data items

The relevant data of the included studies was registered in a standardized Microsoft Excel worksheet. Such data were participant demographics and baseline characteristics, methodology, numbers of sessions and frequency, effect of the intervention measurements at different time intervals, pain level score (the mean and standard deviation of VAS) at baseline, and follow-up intervals. Two reviewers were responsible for data extraction, one reviewer extracted data (RTR), and the other revised the extracted data (LAF). The disagreements were discussed with all reviewers until reaching a consensus. The study researchers were contacted via email for missing data or additional details.

### Risk of Bias in Individual Studies and Quality Assessment

For assessment of the risk of bias of the included studies, the guidelines in Chapter 8 of the Cochrane Handbook for Systematic Reviews of Interventions 10 were followed and Risk of Bias 2 (RoB 2) tool was used to build the graph. 11 Additionally, the quality of each study was evaluated using the Grading of Recommendations Assessment, Development, and Evaluation (GRADE) criteria. 12

## Results

**Fig. 1:**
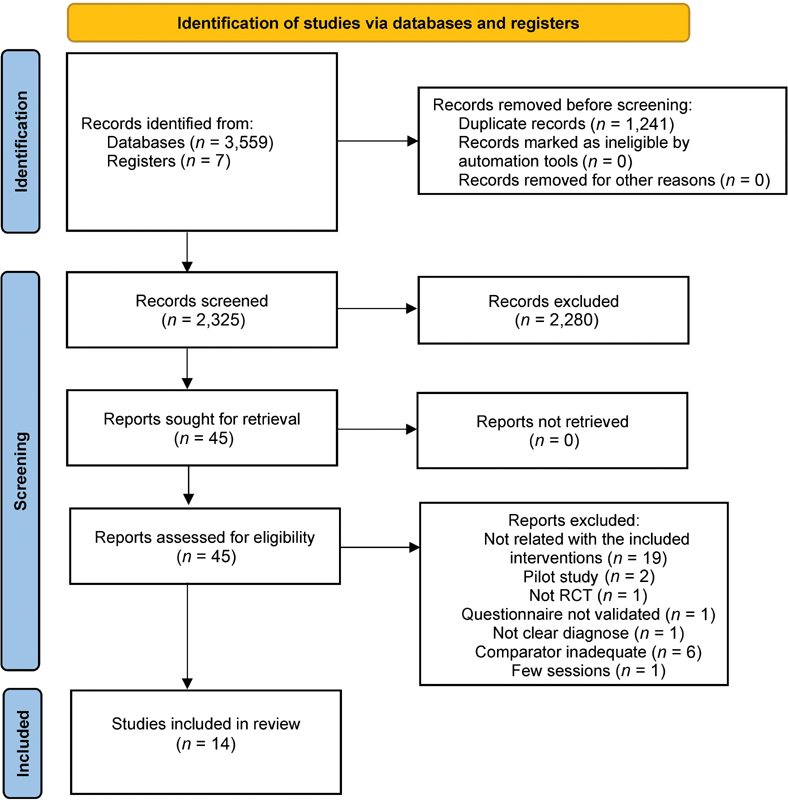
Preferred Reporting Items for Systematic Reviews and Meta-Analyses (PRISMA) flow diagram of the selection process of the studies included in the systematic review. RCT, randomized controlled trial.

**Fig. 2:**
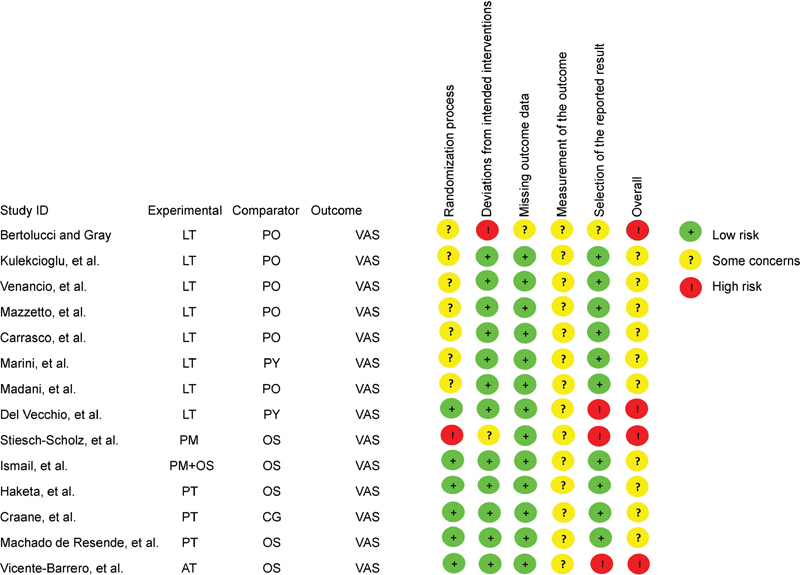
Risk of bias assessment of the included studies. AT, acupuncture therapy; CG, counseling; OS, occlusal splint; PM, physiotherapy by massage; PO, placebo; PT, physiotherapy; PY, pharmacotherapy; VAS, visual analogue scale.

After the search, a total of 3,566 records were found on PubMed ( *n* = 734), Google Scholar ( *n* = 2100), Clinical Trials ( *n* = 7), and Web of Science ( *n* = 725). After removing duplicates, 2325 records remain and were revised by title and abstract. Then, 45 full-text articles were retrieved, of which 31 were excluded with reasons. 13 14 15 16 17 18 19 20 21 22 23 24 25 26 27 28 29 30 31 32 33 34 35 36 37 38 39 40 41 42 43 No gray literature matches the eligibility criteria. The study selection process is detailed in the Preferred Reporting Items for Systematic Reviews and Meta-Analyses (PRISMA) flow diagram ( Fig. 1 ). Finally, 14 articles fully met the eligibility criteria regarding the treatment of pain in TMJD through non-pharmacological interventions (AT, physical therapy, low-level laser, or massage). At the overall bias of the studies included, 71.42% exhibited some concerns and 28.57% had high risk. The main biases were concerns at the measurement of the outcome (100%), concerns in the selection of reported results (28.57%), and concerns in the randomization process (57.14%) ( Fig. 2 ). At the overall quality of the included studies, 21.42% had very low, 50% had low, and 28.57% had moderate certainty of evidence ( Table 2 ). The reasons for not conducting a meta-analysis were the methodological heterogeneity of the interventions as well as the different follow-up intervals found across the studies.

**Table 2:** Quality assessment of the included studies

Certainty assessment							Summary of findings
ID Follow-up of VAS	F1	F2	F3	F4	F5	Overall	Impact
Bertolucci and Grey 48 3 wk	VS a	NV	NS	S c	None	⊕⊕○○ Low	Concerning the symptom and directness of the evidence the finding is critical No more data
Kulekcioglu et al 47 1 mo	S b	NV	NS	S c	None	⊕⊕○○ Low	Concerning the symptom and directness of the evidence the finding is critical No more data
Venancio et al 49 2 mo	S b,	NV	NS	S c	None	⊕⊕○○ Low	Concerning the symptom and directness of the evidence the finding is critical No more data
Mazzetto et al 50 1 mo	S b	NV	NS	NS	None	⊕⊕⊕○ Moderate	The probes factor statistical difference between LT and PO (effective dose, average 2.49306; placebo dose, average 3.2222). Values for evaluations factor showed statistical difference among evaluations (Before treatment = 3.6736; 2 wk = 2.9375; 1 mo = 2.2361; 2 mo = 2.5833) (Turkey's test) Concerning the symptom and directness of the evidence the finding is critical No more data
Carrasco et al 51 2 mo	S b	NV	NS	S c	None	⊕⊕○○ Low	The probes factor without statistical difference between LT and PO (effective dose, average 1.5158; placebo dose, average 4.6507). Multiple comparisons of the evaluations factor showed statistical difference among evaluations (Before treatment = 3.5238; 1 mo = 2.5119; 2 mo = 3.2142) (Turkey's test) Concerning the symptom and directness of the evidence the finding is critical No more data
Marini et al 44 1 mo	S b	NV	NS	NS	None	⊕⊕⊕○ Moderate	The effect of treatment was statistically significant (interaction time-treatment, *p* = 0.0001). Concerning the symptom and directness of the evidence the finding is critical No more data
Madani et al 45 1 mo	S b	NV	NS	VS c , ^d^	None	⊕○○○ Very low	No significant difference in VAS scores between LT and PO ( *p* > 0.05) Concerning the symptom and directness of the evidence the finding is critical No more data
Del Vecchio et al 46 1 wk	VS a	NV	NS	NS	None	⊕⊕○○ Low	Low-level laser therapy was effective (F(2.83) = 4.882; *p* = 0.010) Concerning the symptom and directness of the evidence the finding is critical No more data
Stiesch-Scholz et al 52 Mean 40 mo	VS a	NV	NS	S c	None	⊕○○○ Very low	OS + PM wasn't effective Concerning the symptom and directness of the evidence the finding is critical No more data
Ismail et al 53 3 mo	S b	NV	NS	S c	None	⊕⊕○○ Low	OS + PM wasn't effective. Concerning the symptom and directness of the evidence the finding is critical No more data
Haketa et al 54 8 wk	S b	NV	NS	NS	None	⊕⊕⊕○ Moderate	The changes of Current maximum daily pain intensity in the two treatment groups PT vs. OT *p* -value ^α^ Time progress <0.001; Interaction = 0.12 Concerning the symptom and directness of the evidence the finding is critical No more data
Craane et al 55 52 wk	S b	NV	NS	S c	None	⊕⊕○○ Low	Regression coefficients (β) were not significant. For all outcomes ( *p* > 0.144) Concerning the symptom and directness of the evidence the finding is critical No more data
de Resende, et al 56 1 mo	S b	NV	NS	NS	None	⊕⊕⊕○ Moderate	Significant reduction in patients' pain over time ( *p* < 0.001) in all groups, without relationship between the groups ( *p* = 0.260). This reduction attributed to time was 27.7% ( *ƞ* 2 = 0.277) with a high therapeutic effect Concerning the symptom and directness of the evidence the finding is critical No more data
Vicente-Barrero et al 57 5 wk	VS a	NV	NS	VS c	None	⊕○○○ Very low	AT improved subjective pain, and algometer pressure needed to produce pain. Pain reduction was statistically significant ( *p* < 0.05) Concerning the symptom and directness of the evidence the finding is critical No more data

Abbreviations: F1, factor 1 risk of bias; F2, factor 2 inconsistency; F3, factor 3 indirectness; F4, factor 4 imprecision; F5, factor 5 publication bias; NS, not serious; NV, not evaluable/single study; S, serious; SADG, strong association dose response gradient; VS, very serious.

Explanations:

a High risk in the bias assessment.

b Some concerns in the risk of bias assessment.

c Small groups.

### Laser Treatment

Three of the included studies recruited participants according to the DC/TMD guideline 44 45 46 and five studies recruited them according to criteria compatible with the DC/TMD for the diagnosis of TMJD. Four of the included studies assessed all participants with additional imaging through magnetic resonance imaging scan 44 47 or cone-beam computed tomography ( Table 3 ). 45 46

**Table 3:** Individual characteristics of the included studies with laser intervention for pain relief in TMD

Study	Population	Intervention/comparator	Outcome/Result	Direction of effect
Bertolucci and Grey 48	Patients with degenerative joint disease ( *n* = 32)	G1: LT G2: PO	Mean VAS Change: G1: 40.25 ± 1.56 G2: 1.56 ± 8.57	LT vs. PO Favors LT
Kulekcioglu et al 47	Joint disorders ( *n* = 18) Muscle disorders ( *n* = 17)	G1: LT ( *n* = 20) G2: PO ( *n* = 15)	Mean VAS values Baseline: G1: 42.8 ± 27.0 G2: 35.3 ± 29.0 After treatment: G1: 10.5 ± 18.5 G2: 8.0 ± 9.4 1 mo after treatment: G1: 5.5 ± 19.7 G2: 5.3 ± 6.4	LT vs. PO a No effect
Venancio et al 49	Joint disorders ( *n* = 30)	G1: LT ( *n* = 15) G2: PO ( *n* = 15)	Mean VAS values Baseline: G1: 8.27 ± 1.79 G2: 7.73 ± 1.91 60 d after treatment: G1: 1.6 ± 2.03 G2: 7.73 ± 1.91	LT vs. PO a No effect
Mazzetto et al 50	Joint disorders ( *n* = 48)	G1: LT ( *n* = 24) G2: PO ( *n* = 24)	Analysis of variance and Turkey's test: G1: 2.49306 G2: 3.22222 Critical value: 0.62957	LT vs. PO Favors LT
Carrasco et al 51	Joint disorders ( *n* = 40)	G1: LT ( *n* = 20) G2: PO ( *n* = 20)	Analysis of variance and Turkey's test: G1: 1.5158 G2: 4.6507 Critical value: 0.73459	LT vs. PO Favors LT
Marini et al 44	Joint disorders ( *n* = 99) Patients with disc displacement without reduction ( *n* = 30) Patients with osteoarthrosis ( *n* = 69) Patients with intraarticular effusion ( *n* = 79)	G1: LT ( *n* = 39) G2: PY ( *n* = 30) G2: PO ( *n* = 30)	Mean VAS values Baseline: G1: 7.72 ± 0.41 G2: 7.42 ± 0.51 G3: 7.13 ± 0.88 At day 15: G1: 0.07 ± 0.13 G2: 6.36 ± 1.16 G3: 6.09 ± 0.94	LT vs. PY Favors LT
Madani et al 45	Patients with osteoarthritis ( *n* = 20)	G1: LT ( *n* = 10) G2: PO ( *n* = 10)	Percentage of improvement: G1: 48% G2: 32%	LT vs. PO a No effect
Del Vecchio et al 46	Patients with mixed joint disorders	Laser (LT) ( *n* = 30) Placebo (PO) ( *n* = 30) Pharmacotherapy (PY) ( *n* = 30)	Mean VAS at T0 and T1 LT: T0 65.52 ± 17.441 T1 30.34 ± 20.439 PO: T0 58.57 ± 15.567 T1 36.43 ± 21.294 PY: T0 74.48 ± 13.252 T1 37.59 ± 23.092	LT vs. PO Favors LT LT vs. PY a No effect

Abbreviations: CL, control; LT1, laser 1; LT2, laser 2; PO, placebo; PY, pharmacotherapy; TMD, temporomandibular disorder; VAS, visual analogue scale.

Standard deviation (±).

a Not statistical difference between groups.

Bertolucci and Grey 48 evaluated the effect of nine sessions during 3 weeks of LLLT. In the LLLT group, the device applied infrared radiation (at 904 nm wavelength, 700 Hz, 27 W, for 9 minutes, and 100% of power output, no more data). The comparator was PO with the laser device switched on, but not working. The results showed a statistical difference in favor of the intervention ( *p* < 0.01). The researchers also evaluated biomechanics (total vertical opening and lateral deviation) reporting a statistical difference in favor of the LLLT ( *p* < 0.01).

Kulekcioglu et al 47 evaluated 12 sessions of LLLT applied during 1 month. The intervention with a gallium-aluminum-arsenide (GaA1As) diode laser (at 904 nm of wavelength, 1000 Hz, for 180 seconds 3 J/cm ^2^ ) was applied to the most tender points; on the other hand, the comparator was PO. The reported results showed a significant reduction of the pain in both groups, without statistical differences between groups ( *p* = 0.438). Also, the researchers evaluated other variables, reporting a statistical difference in favor of the LLLT, as tender points ( *p* = 0.001), active mouth opening ( *p* = 0.001), passive mouth opening ( *p* = 0.003), right lateral motion ( *p* = 0.005), and left lateral motion ( *p* = 0.002).

Venancio et al 49 assessed six sessions of LLLT applied for 3 weeks with a GaA1As (at 780 nm of wavelength, 30 mW, for 10 seconds and 6.3 J/cm ^2^ ) diode laser. LLLT was administrated at three TMJ points ( the posterior area of the joint with the mouth open; the area anterior to condyle in the sigmoid notch with the mouth closed; the joint interface with the mouth open). The comparator was PO with an inactive laser device. The results showed a decrease in pain intensity ( *p* < 0.001) in both groups comparing the baseline and the end VAS (at 60 days); however, the results showed no statistical difference between the groups ( *p* = 0.05). Also, the researchers evaluated painless maximum vertical opening (MVO), right (RLE) and left lateral excursion (LLE), and protrusion excursion (PE). The results showed no statistical differences between groups (MVO *p* = 0.20, RLE *p* = 0.29, LLE *p* = 0.32, and PE *p* = 0.70).

Mazzetto et al 50 evaluated the effect of eight LLLT sessions applied for 4 weeks. The intervention was performed with GaA1As (at 780 nm of the wavelength, 70 mW, for 10 seconds and 89.7 J/cm ^2^ ) diode laser. The LLLT was applied in continuous mode and in contact with the skin at a point located above the external auditive canal toward the retrodiscal region. The comparator was PO with an inactive laser device. The results showed statistical differences in VAS between groups in favor of LT. Later the same research team Carrasco et al 51 in a similar protocol of LT evaluated a dose of 105 J/cm ^2^ applied in five points (located within the TMJ area). The results showed statistical differences in VAS between groups in favor of the LT.

Marini et al 44 evaluated 10 consecutive sessions of LLLT in patients with TMJ pain. The device used was a GaA1As with a wavelength of 780 nm with time pulsation < 200 nanoseconds, frequency range of 1 to 50 kHz, mean power of 400mW, and a peak power 45 W diode laser. The LLLT were 20 kHz for 10 minutes, 18 kHz for 5 minutes, and 16 kHz for 5 minutes (no more data). The comparators were the PY group (800mg twice a day of ibuprofen for 10 days) and the PO group using only red light of the laser without energy for 20 minutes. The results showed a statistical difference in favor of the LLLT ( *p* < 0.001). Also, the researchers evaluated active mouth opening, passive mouth opening, right lateral motion, left lateral motion and reported a statistical difference in favor of the LLLT ( *p* = 0.001).

Madani et al 45 evaluated the effect of 12 sessions for 4 weeks of an infrared LLLT (at 810 nm of wavelength, with a 50 mW average power at a pulse repetition rate of 1500 Hz, pulse length of 1 millisecond, 6 J per point, 3.4 J/cm2, and spot size 1.76 cm2) applied for 2 minutes per point. The total dose was in a range of 27.2 to 60.8 J/cm ^2^ . The control group was PO with the same sessions without laser irradiation. The results showed no significant difference in the pain of the masticatory muscles between the two groups ( *p* > 0.05).

Del Vecchio et al 46 evaluated 14 sessions of LLLT, twice a day for 7 consecutive days in patients with TMJ pain. The LLLT (at 808 nm of wavelength, dose at 5 J/min, 250 mW, and 15 kHz for 8 minutes, for a total of 40 J each) was applied directly over the pain area. The comparators were two groups: PO with sham laser and PY (two nonconsecutive cycles of nimesulide 100 mg per day for 5 days, interspersed with one cycle of cyclobenzaprine hydrochloride 10 mg per day for 5 days). The authors found no difference between the LLLT and PY regarding the pain level registered.

### Massage and Physiotherapy

We have identified five studies that matched the eligibility criteria of the review; two articles with massage or manual therapy (MT) 52 53 and two articles with PT 54 55 56 met inclusion criteria ( Table 4 ). Stiesch-Scholz et al 52 and Ismail et al 53 evaluated the effect in TMJD of OS plus MT to mobilize the TMJ. The patients were treated for 30 52 or 45 53 minutes twice a week. The comparator was the OS alone used for 24 hours a day, excluding meal times. Regarding pain relief, Stiesch-Scholz et al 52 reported that the control group had greater pain relief, with a mean follow-up of 40 months. Conversely, Ismail et al 53 reported no significant difference between the groups after 12 weeks of the end of the treatment.

**Table 4:** Individual characteristics of the included studies with physiotherapy or massage intervention for pain relief in TMD

Study	Population	Intervention/comparator	Outcome/Result	Direction of effect
Stiesch-Scholz et al 52	Patients with anterior disc displacement without reduction ( *n* = 72)	G1: OS ( *n* = 21) G2: OS + PY ( *n* = 24) G3: OS + PM ( *n* = 7) G4: OS + PY + PM ( *n* = 20)	Pain-symptoms after therapy: G1: 10% Unchanged 14% Improved 76% Pain free G2: 12% Improved 88% Pain free G3: 28% Unchanged 29% Improved 43% Pain free G4: 10% Unchanged 25% Improved 65% Pain free	Favors OS + PY
Ismail et al 53	Patients with disc displacement without reduction ( *n* = 21) Patients with disc displacement with reduction ( *n* = 3) Patients with osteoarthrosis ( *n* = 2)	G1: OS ( *n* = 13) G2: OS + PM ( *n* = 13)	Total pain intensity: G1: Δ 23 ± 22 G2: Δ 28 ± 21 Pain intensity during mandibular movement: G1: Δ 25 ± 22 G2: Δ 23 ± 27 Pain intensity without mandibular movement: G1: Δ 8 ± 9 G2: Δ 16 ± 17 Pain intensity after mandibular loading: G1: Δ 36 ± 25 G2: Δ 49 ± 35	a No effect
Haketa et al 54	Patients with disc displacement without reduction ( *n* = 52)	G1: PT ( *n* = 19) G2: OS ( *n* = 25)	Mean VAS values Baseline: G1: 63.1 ± 28.02 G2: 58.9 ± 28.2 After 4 wk: G1: 33.1 ± 26.8 G2: 43.5 ± 27.1 After 8 wk G1: 21.3 ± 26.4 G2: 36.5 ± 28.7	a No effect
Craane et al 55	Patients with disc displacement without reduction ( *n* = 49)	G1: PT ( *n* = 20) G2: CG ( *n* = 22)	Median (25th- 75th percentile) VAS values Baseline: G1: 50 (38–60) G2: 54.5 (40–65) After 52 wk: G1: 2 (0–16) G2: 2.5 (0–13)	a No effect
de Resende et al 56	Mixed TMD ( *n* = 89)	G1: PT ( *n* = 21) G2: OS ( *n* = 22) G3: CG ( *n* = 17) G4: OS + CG ( *n* = 25)	Mean VAS values Baseline: G1: 3.43 ± 2.18, G2: 3.50 ± 3.11, G3: 5.00 ± 2.59, G4: 4.68 ± 2.97 After 30 d: G1: 1.16 ± 2.19, G2: 1.82 ± 1.65, G3: 4.41 ± 3.08, G4: 2.52 ± 2.62	a No effect

Abbreviations: CG, counseling; OS, occlusal splint; PM, physiotherapy by massage; PO, placebo; PT, physiotherapy; PY, pharmacotherapy; TMD, temporomandibular disorder; VAS, visual analogue scale.

Standard deviation (±)

a Not statistical difference between groups.

Haketa et al 54 evaluated 8 weeks of PT. The patients performed four sets per day of exercises that included stretching movements of the jaw. The comparator was OS; both groups were prescribed amfenac sodium 3 times every day. The team reported that daily pain intensity significantly decreased in both treatment groups, and but no additional beneficial effect of PT was found.

Craane et al 55 evaluated 6 weeks of PT. The home exercise program included cheeks and tongue in rest position, active mouth opening exercises, use of cold or hot packs, and self-massages. Besides, the patients had nine treatment sessions of physical therapy. The comparator was counseling, the team reported that there was no significant difference between the groups at the 52nd week. Also, the researchers evaluated active mouth opening and passive mouth opening resulting in favor of PT ( *p* = 0.03; *p* = 0.004). Conversely, the mandibular function impairment questionnaire results exhibited no significant difference between groups.

de Resende et al 56 assessed the outcomes of PT with 4 weeks of exercises with a trainer researcher, each session lasted 40 minutes for a total of eight sessions. The intervention also included the application of warm compresses (40–50°C) for 20 minutes three times per day and 10 minutes of massage on the masseter and temporalis muscles at home. This intervention differed from normal practice, due to combining assisted PT and self-massage. The comparators were OS, counseling (CG), and OS plus CG. Their results showed a statistically significant reduction in pain over time ( *p* < 0.001) in all groups; however, there was no a significant difference between them ( *p* = 0.260). Also, the authors evaluated sleep quality(PSQI), the impact of oral health on quality of life (OHIP-14), and quality of life using the WHOQOL questionnaire (QL). Nonetheless, the results showed no significant differences between the groups.

### Acupuncture

Vicente-Barrero et al 57 evaluated the effect of AT for pain relief. Treatment consisted of 15 sessions of 30 minutes each, for 5 weeks. The needles were placed in the points EXHN5, SJ21, GB2, SJ17, ST6, LI4, ST36, SJ5, and GB34. The control group received OS. The researchers reported the pain results in favor of the AT. Also, the researchers evaluated the mouth opening and reported statistical difference in favor of the AT ( *p* < 0.05). No further articles were found using the AT intervention for pain relief of the TMJD according to the eligibility criteria of this review ( Table 5 ).

**Table 5:** Individual characteristics of the included studies with acupuncture intervention for pain relief in TMD

Study	Population	Intervention/comparator	Outcome/Result	Direction of effect
Vicente-Barrero et al 57	Joint disorders ( *n* = 18)	G1: AT ( *n* = 10) G2: OS ( *n* = 10)	VAS reduction Basale vs. Day 30 G1: ( *p* < 0–05) G2: ( *p* > 0–05) No more data.	Favors AT

Abbreviations: AT, Acupuncture therapy; G, group; OS, occlusal splint; TMD, temporomandibular disorder; VAS, visual analogue scale.

## Discussion

In the present review, we found a low quality of evidence across the studies. However, the evidence suggests that the LLLT, among the interventions presented in this article, showed a high effect as a nonpharmacological intervention for treating painful TMJD. Some reports have been postulated that laser biostimulation influences cellular metabolic processes that promote analgesic, anti-inflammatory, and healing effects. In consequence, LT were introduced more than four decades ago to treat various conditions in which pain plays a significant role in the clinical conditions studied. However, until 2019, the US Food and Drug Administration clears LT for chronic musculoskeletal pain. 58 Nonetheless, the included studies had a considerable diversity in their treatment plan with differences in irradiation (wavelength, energy density, and spot area), stimulation areas, sessions, and follow-up time. Also, the studies had problems with the randomization process, selection of patients and samples sizes, subjective pain measurements, and report selection. In consequence, low quality of the evidence and some concerns in bias were found. Other limitations on the included studies relied on the lack of stratified sampling of the patients according to DC/TMD Axis I diagnostic criteria to compare the efficacy of the interventions.

To our knowledge, the published systematic reviews of the treatment of painful TMJD with studies that using LT are focused on masticatory muscle disorders or included multiples pathologies (TMD and TMJD combined) without reported the results of each one separately. A systematic review concerning TMD treatment by Chen et al 59 included 14 trials for the outcomes pain (VAS), active and passive AM. Their meta-analysis concluded that LT has limited efficacy for pain relief; however, this analysis exhibited a high degree of heterogeneity between the pooled studies. Moreover, there was a lack of definition of the treatment plan (laser irradiation) in several studies included. Recently, Ahmad et al 60 included 31 LT trials for the treatment of TMD, and the data were pooled according to LT dosage. Their meta-analysis showed a significant difference between the LT and PO groups; however, a high heterogeneity (I ^2^ = 90%) was exhibited. This heterogeneity is probably attributable to the diversity of disorders assessed in the included studies and may be meaningless. Further, the studies in the meta-analysis had only PO as a control group. This approach of comparing the effect of an intervention with a PO (untreated control group) leads to overestimating the treatment effect, so the control group should be treated with a standard treatment rather than nothing. 61

The present review focuses on TMJD to decrease heterogeneity. However, the included studies showed great diversity in laser dosing, which explains the impossibility of conducting a meta-analysis. Also, most of the studies use PO as comparator. Still, future research will be focused on the components that might influence the efficacy of LLLT (wavelength, energy density, spot area, dose, optimal applied points, and time of application). Also, further research should include appropriate power analysis, reliability, validity (internal and external), adequate comparators, and responsiveness of outcomes assessed.

Concerning PT, in the present systematic review, we find positive results when using exercises for TMJD, but no statistical differences were found comparing OS or CG. Also, the included randomized clinical trials (RCTs) had great diversity in their treatment plan. The activities prescribed were combined with massage or the use of warm compresses. These make it difficult to conclude that there is a benefit of using PT. On the other hand, MT showed no improvement in managing pain in TMJD. In 2016, Armijo-Olivo et al 62 conducted a systematic review. The aim was to compare any manual intervention as in mobilization, manipulation, or exercise therapy alone for the treatment of TMD. In their results, these exercises, compared with control groups without treatment, showed improvement at the function, decreasing joint pain and pain sensitivity of the masticatory muscles. However, the differences between exercises and other forms of active treatments (splints, global postural re-education program, or AT) were not significant. Besides, the overall quality of the evidence was low. Theoretically, the physical forces generated during the exercises can induce a complex interactive biological network. This involves the musculoskeletal structures that, in response to these stimuli, transduce biochemical signals, and influence each tissue configuration to physiological recovery adapting their resistive forces. It is a physical strain that modulates the activation of diverse cells, along with their lineage commitment and maturation. The clinical result of physical exercise is high bone and muscle mass, resulting in enhanced tissue resistance allowing physiological recovery. 63 A plausible improvement to the design of RCTs in future research on LT should be to avoid short-term treatment. Changes in bone density secondary to exercises have been reported up to 6 months after PT. Also, the changes in the joint fluid can be seen after 3 months of exercise protocols. 64 So, a shorter treatment may be ineffective due to insufficient time and not because of the intervention itself. 65

In this systematic review, only one clinical trial was found using AT to treat pain in TMJD. The study performed by Vicente-Barrero et al 57 had a high risk of bias and very low quality of evidence. Despite that AT is an effective complementary therapy for treating the pain and inflammation in multiple disorders such as arthritis, headache, posttraumatic stress disorder, colitis, and postoperative recovery. 66 67 68 69 The efficacy of this therapy remains controversial. Basic research has demostrated, in animal models and some human pathologies, that AT can modulate pro-inflammatory cytokines. This anti-inflammatory effect depends on the neuronal networks and their mechanisms of neuroimmunomodulation. 70 When the needle is inserted in an acupuncture point, the peripherical nerve system is stimulated due to a nerve depolarization, and in consequence, there is a release of neuropeptides, hormones, 71 and neurotransmitters, such as acetylcholine and catecholamines. 72 73 These mediators can modulate the stress, inflammation (cell activation, differentiation, and immunophenotype), 74 and pain. Such conditions have been linked to various types of diseases in a variety of medical disciplines, for instance, in dentistry. 75 However, a previous review was conducted by Jung et al 76 with articles until July 2010 and concluded that the evidence for AT as a symptomatic treatment of TMD is limited. Fernandes et al 77 published an AT review including records from 1990 to May 2015. The team concluded that the articles showed low methodological quality and the reliability of the evidence was limited to support the AT for the treatment of TMD. Other researchers have similar conclusions so far. 78 Also recently, Al-Moraissi et al 79 conducted a meta-analysis of RCTs; the aim was to compare wet needling, dry needling, and AC for pain treatment in TMD. Their results showed that there is not enough evidence to support any of the needling therapies for TMD. In consequence, AT intervention requires more controlled and RCTs with larger sample sizes and high quality to verify their potential use in the different classifications of TMD.

PY had been used to treat TMD including the prescription of nonsteroidal anti-inflammatory drugs, opioids, corticosteroids, anxiolytics, muscle relaxants, benzodiazepines, and even antidepressants. Diverse application techniques to improve the pharmacokinetics and pharmacodynamics of drugs have emerged. In that sense, nanotechnology has been used as a novel approach for dentistry treatments, 80 81 82 83 and due to that, nanocarriers have been developed for TMD treatments. 84 However, there is not enough evidence to confirm which drugs are effective for reducing painful TMD. 85 In consequence, there is still a need to find adequate complementary treatment.

### Study limitations

A single RCT of AT, the relatively low number of RCTs addressing the isolated effect of PT techniques, the heterogeneity of the interventions in the PT and LLLTs studies impairs the synthesis of evidence regarding the different nonpharmacological treatments. Moreover, the studies showed large methodological differences in the study design, in the duration of the prescription, frequency of the procedure, and follow-up. There is a need for high-quality RCTs addressing the best regimen of PT and LLLTs with adequate study design and application of standardized methods to evaluate the subjects to improve clinical evidence on the effectiveness of nonpharmacological interventions.

## Conclusions

### Research Implications

The overall quality of evidence of nonpharmacological treatments was low showing that there is lack of certainty about these therapies as options for the pain-relieving in TMJD. There is a clear need for well-designed RCT examining the nonpharmacological treatments for TMJD. PT trials must be performed, confining the type of exercise in the PT that is under proving to allow knowledge of the effectiveness of this treatment. Besides, more details of activity, dosage, and frequency should be reported to create reproducible results. Also, reports of complete results and statistical analysis are required. LT trials must address on the best prescription (wavelength, energy density, spot area, dose, optimal applied points, time of application) for painful TMJD treatment.
